# Supplementary material for: Ballistic, maximal strength and strength-endurance performance of male handball players: Are they affected by the evaluator’s sex?
Source: PLoS One. 2021 Apr 22;16(4):e0249974. doi: 10.1371/journal.pone.0249974 (PMC8062097; doi:10.1371/journal.pone.0249974)
Supplement: Attachment — Submitted filename: Review of Manuscript_PONE.docx [file pone.0249974.s002.docx]

Review of Manuscript:

Ballistic, maximal strength and strength-endurance performance of male handball players: are they affected by the evaluator's sex?

Overall, this is a well-written manuscript that may contribute to the aims of PlosOne and to relevant scientific literature. A paucity of work has explored the impact of evaluator sex on exercise testing outcomes. This study was designed to extend work in this domain and offers novel directions for continued research. There are certain concerns that I feel should be addressed to shift this paper closer to publication readiness.

1. In addition to social facilitation, some research brings up the converse phenomenon of social loafing. I think it is worth highlighting this distinction, particularly as social loafing is often observed in group contexts when individuals are working collectively towards a goal, and other male handball players (of the same team affiliation) were present during testing.

2. It is not a flaw of the study, but a clear justification of why the impact of evaluator sex on female handball players’ performance was not evaluated is needed.

3. Evidence of a sample size estimation should be provided to ensure that this study was adequately powered to detect an experimental effect.

4. On lines 115-116, I am confused by the heading “Experimental Design,” which is followed by the text “a cross-sectional study was designed.” Cross-sectional research is non-experimental because the groups are not randomly selected, and the independent variable is not manipulated. Please revise to clarify.

5. Additional information about the male and female evaluators would be helpful.

- What were the ages of the evaluators?

- Did the evaluators know any of the participants or vice-versa?

- Why did you choose to have the evaluators adopt a test-administration role, rather than a passive observer, or a (potentially) supportive role as a spotter? It is important to justify this decision because the dynamic of authority could arguably have an impact on handball players’ perceptions of evaluators. You also emphasize this test-administrator/passive observer dichotomy in the discussion, so additional elaboration is warranted.

6. One concern is that there was no inclusion of a control group. That is, we cannot confirm that the presence of female evaluators is meaningfully associated with male handball player BP performance, relative to the presence of male evaluators. It could be that unmeasured variables impacted study outcomes. A control group with no evaluators would be the most ideal to establish a baseline index of performance. However, if participant safety or protocol adherence is a concern, such that participants cannot be tested alone, then another approach may be to employ mixed-sex evaluation in a separate condition. If significant effects were to emerge with same sex evaluation compared to simultaneous evaluations conducted by one male and one female evaluator, then this evidence may be more convincing.

On lines 268-270, you write, “Given that in the present study only 1 out of 15 correlations reached statistical significance, it is likely that the significant correlation may be anecdotal and promoted type I error.” What statistical adjustments did you use to correct for multiple comparisons? If you are concerned that Type 1 errors are a threat to the validity of your conclusions, what is the rationale for running these analyses?

7. Why were other members of the handball team present during testing? It seems like the study may be confounded by this. Though it is highlighted as a limitation, additional explanation for this decision should be provided.

8. Given the lack of control group and the presence of the handball team during exercise testing, I do not think that the following claim can be made: “The results of the present study collectively suggest that the sex of the evaluator is not an important factor to consider when assessing strength performance in young male athletes. Therefore, male handball players can be indistinctly assessed by male or female evaluators.” Though your study suggests that the majority of physical outcomes were not differentially impacted by male or female evaluation, more research is needed before we can confidently conclude that male handball players’ performances may not be significantly affected by the sex of evaluators.

Minor comments:

On line 77, I think “adversary” should be changed to adversaries

On line 288, I think “presented” should be changed to present
